# Canine gastrointestinal parasites perceptions, practices, and behaviours: A survey of dog owners in Australia

**DOI:** 10.1016/j.onehlt.2023.100587

**Published:** 2023-06-16

**Authors:** Luca Massetti, Rebecca J. Traub, Louise Rae, Vito Colella, Lara Marwedel, Phillip McDonagh, Anke Wiethoelter

**Affiliations:** a Melbourne Veterinary School, Faculty of Science, University of Melbourne, Parkville, VIC 3052, Australia; b Boehringer Ingelheim Animal Health Australia, North Ryde, New South Wales 2113, Australia

**Keywords:** Intestinal worms, Questionnaire, Pet owners, Parasite control, Deworming

## Abstract

Many species of canine gastrointestinal (GI) parasites are known to be zoonotic meaning that dog owners' management and practices are key to preventing exposure of humans and dogs as well as contamination of the environment. As Australia has one of the highest rates of pet ownership in the world, we administered an online questionnaire to dog owners across the nation to assess their perceptions, practices, and behaviours towards canine GI parasites. Descriptive analysis was performed to summarise perceptions and management practices. Factors associated with the suitability of parasiticide treatments applied were investigated using uni- and multivariable ordinal regression. Just over a half of dog owners considered parasites as very or extremely important for their dog's health (59%) and less than a half as very or extremely important for human health (46%). Although the majority of dog owners stated that they deworm their dogs (90%), only the 28% followed best practice guidelines, i.e. administered a monthly prophylactic treatment all-year round. A large proportion of respondent dog owners administered prophylactic treatment at an inappropriate frequency (48%) or did not treat for canine GI parasites at all (24%).

Attending vet visits at least once a year or once every six months and having a very comfortable or prosperous financial position were significantly associated with following best deworming prophylaxis guidelines. This study demonstrates that a proportion of dog owners in Australia is not complying with best practice regarding the control of canine GI parasites and is potentially exposing themselves and their dogs to the risk of infections. Veterinarians are called to implement dog owner's education, raise their awareness on the threats canine parasitic diseases pose to both dogs and humans and finally, encourage them to follow a monthly prophylactic treatment for canine GI parasites all year round.

## Highlights

• Just half of dog owners in Australia considered canine parasites as very or extremely important.
• The majority of dog owners in Australia did not follow best deworming practice guidelines.
• Socioeconomic factors and vet visits are associated with following best deworming practice.

## 1 Introduction

Australia has one of the highest rates of pet ownership in the world, with almost two-thirds of households owning pets of which, dogs are the most commonly owned with an estimated population of approximately 6.3. million [1]. Pet ownership has been correlated to better human physical and psychological health with benefits in pet owners such as reduction of blood pressure, stress and anxiety resulting in a reduced risk of diseases such as mental health disorders and coronary heart disease reported [[2], [3], [4]]. However, pet ownership may also increase the risk of exposure of humans to zoonotic pathogens including canine endoparasites [[5], [6], [7]].

The last nationwide canine endoparasite survey conducted in Australia over 10 years ago found at least 14 parasites infecting dogs nationally, namely, *Giardia duodenalis*, *Ancylostoma caninum*, *Ancylostoma ceylanicum*, *Uncinaria stenocephala, Toxocara canis, Toxascaris leonina, Taenia* spp.*, Echinococcus granulosus, Spirometra erinacei*, *Dipylidium caninum, Trichuris vulpis, Cystoisospora* spp., *Cryptosporidium* spp., and *Sarcocystis* spp., the majority of which are potentially zoonotic [8]. More recently, a national study on the contamination of urban dog parks with canine soil-transmitted helminths (cSTHs) in Australia highlighted the high risk of exposure of dogs and their owners to cSTHs and in particular to hookworms, *Strongyloides* spp. and *Trichuris* spp., the former two having proven zoonotic potential [9].

The husbandry and management practices of dog owners can predispose their dogs to parasitic infections and increase their own risk of exposure. For example, failing to clean up dog faeces on their premises, feeding raw meat diets, housing pets outdoors, and a lack of or ineffective anthelmintic treatments have been linked to significantly higher rates of gastrointestinal (GI) parasite infections in pet and working dogs [8,[10], [11], [12]]. Matos et al. (2015) found that even though most pet owners in Portugal administer anthelmintic drugs to their dogs, this often occurs at irregular intervals and as a consequence, the treatment is often inefficient. Furthermore, the frequent use of obsolete active ingredients and incorrect treatment protocols may not only fail in killing the parasites but may also increase anthelmintic resistance [14]. A large-scale survey of the practices and behaviours of dog owners in Australia in relation to canine endoparasites found that although the majority of dog owners dewormed their dogs, 34% relied on products based on pyrantel embonate alone, which in regard to nematodes is not efficacious against whipworm (Australian Pesticides and Veterinary Medicines Authority, www.apvma.gov.au.) and for which resistant strains of *A. caninum* have been reported in Australia [14].

More recently, a study of dog owners' perceptions towards canine GI parasitism and associated human health risks performed in Southeast Queensland found that dog owners' knowledge plays a key-role in preventing parasitoses among dogs, with dog owners aware of the threats canine GI parasites posed more likely to deworm their dogs and adequately dispose of dog waste [15].

In the current study, we surveyed dog owners across Australia, to update and establish on a national scale, dog owners' perceptions, practices, and behaviour, towards canine GI parasites. In doing so, we aimed to identify potential risk factors that may increase the risk of transmission of these parasites to dogs and their owner. Through this, we aim to inform veterinarians on how to best target dog owner education for the improvement of animal and human health.

## 2 Materials and methods

### 2.1 Ethical statement

This study was approved by the Human Ethics committee of the University of Melbourne; Ethics ID number 1954758.2. Prior to participation, informed consent to use the data gathered in this study was obtained from all participants. Participants were informed on the aims and the methods of this study before completing the questionnaire. Participation in this study was voluntary and anonymous.

### 2.2 Questionnaires

Between February 2020 and October 2021, online questionnaires were administered to dog owners currently residing in Australia. The online questionnaire was designed in Research Electronic Data Capture (REDCap) [16], which supports mobile devices as well as computers. The questionnaire was based on a previously published questionnaire on practices and perceptions of dog owners in Australia towards canine GI parasites [17]. This questionnaire was extended to include further details on deworming protocols in line with literature reviewed [[18], [19], [20]] and dog owners demographics based on a nationally longitudinal study of Australian households [21]. The questionnaire was piloted with ten dog owners and revised based on their feedback received, before being distributed via QR codes on flyers in dog parks, pet shops, veterinary clinics as well as through social media (Facebook) and PureProfile (Surry Hills, NSW, Australia). The questionnaire consisted of three sections: 1) respondent's demographics 2) respondent's perceptions and management of canine GI parasites, and 3) demographics of the respondent's dogs (Supplementary material S1).

Respondent demographic information covered gender, age, postcode, financial situation, and level of education. Information on the awareness and perceptions of canine GI parasites was gathered by asking owners how problematic they perceived parasitic diseases being for their dog and for their own health, ranked on a 5-point Likert scale with 1 being “Not at all” and 5 being “Extremely”. Information on practices relevant to the risk of exposure to, and methods of control of canine GI parasites was gathered though a series of questions investigating the dog's diet, housing, walks, frequency of vet visits, contact with other animals, and treatment for canine GI parasites, including products used and frequency of administration. Finally, basic signalment such as dog's age, weight, sex, and breed was obtained. Dog owners with multiple dogs were asked to include only one in the study.

### 2.3 Data analysis

At completion of the survey period, all participant responses were exported into SPSS for Windows Version 27 (SPSS Inc./IBM, Chicago, Illinois, USA). Responses were checked for plausibility, duplicates, and completeness. Questionnaires with <75% of the questions answered were considered as incomplete and excluded from further analysis.

The outcome variable, named suitability of parasiticides protocol, was created based on the frequency owners administered products effective against canine GI parasites. Dog owners that treated for canine intestinal parasite at the recommended monthly frequency all year around were considered to follow best prophylactic practice according to established guidelines (appropriate prophylactic treatment) [19,20]; dog owners who administered an effective prophylactic treatment at a longer interval (e.g. every 2–3 months or 4–6 months) or did not treat all year round, were considered not to follow best prophylaxis guidelines (inappropriate prophylactic treatment). Dog owners who claimed not to administer any prophylactic treatment or gave products ineffective against canine GI parasites were considered not to follow any effective prophylaxis treatment (no prophylactic treatment).

Explanatory variables were assessed by creating frequency tables and bar charts. Dog breeds were categorized into their corresponding group based on the classification of the Australian National Kennel Council (https://dogsaustralia.org.au/members/Breed/Index/1). Contingency tables of explanatory variables with the three-level outcome variable (appropriate prophylactic treatment, inappropriate prophylactic treatment, no prophylactic treatment) were created and examined for small numbers. Where appropriate, explanatory variables were re-categorized: postcodes were summarized by state into “New South Wales”, “Victoria”, “Queensland”, and “Other”; age into“18–34”, “35–54”, and “over 55 years old”; financial situation into “Finding it difficult or just getting along”, “Reasonably comfortable”, and “Very comfortable or prosperous”; area of residence into “Inner city”, “Suburbia/Semi-rural”, and “Rural/ Remote”, and perceived importance of parasites on dogs and humans' health into “Not at all/little”, “Moderately”, and “Very/Extremely”.

Associations between suitability of parasiticide protocol and respondent's gender, age, financial situation, state, area of residence, perceived importance of parasites on dog's health, perceived importance of dog's parasites on human health, and dog's age, sex, weight, breed, number of dogs per household and frequency of vet visits, was investigated using uni- and multivariable ordinal regression analyses. Variables with a *p*-value ≤0.25 in the univariable analysis were considered eligible for inclusion in the multivariable ordinal regression analysis. Multicollinearity was checked using Variance Inflation Factors (VIF), Pearson correlation coefficient for continuous variables and Spearman's rank-order correlation for categorical variables. Variables with a coefficient >0.6 were considered highly correlated and only one of the pair was included in the analysis.

For the multivariable regression analysis, a strategy of backwards stepwise variable selection was used where the least significant variable (*p*-value ≤0.05) was removed at each step to derive the final model. Potential confounding variables were added one-by-one back into the final model and the change of regression coefficients was examined with a change of >20% deemed meaningful. Model fit was assessed using goodness of fit tests. The proportional odds assumption was tested using the Score test.

## 3 Results

In total, 814 survey responses were recorded, however only 759 were complete (93%). Demographics of dog owners are shown in Table 1. The majority of participating dog owners were females (73%), between 35 and 54 years of age (43%), with either a Certificate IV or below (39%) or Diploma/Advanced Diploma/Bachelor's degree (38%), and resided in the state of Victoria (34%) or New South Wales (24%), in suburbia/semirural areas (81%).

**Table 1:** Demographics of Australian dog owners responding to an online survey on perceptions, practices and behaviour regarding canine gastrointestinal parasites (*n* = 759).

Variable name	Categories	N of respondents	Frequency	Percentage (%)
Sex		752		
	Female		545	72.5
	Male		207	27.5
Age		756		
	18–34 years		222	29.4
	35–54 years		321	42.5
	≥55 years		213	28.2
Education		755		
	Certificate IV or below		294	38.9
	Diploma/ Advanced Diploma/ Bachelor's degree		289	38.3
	Graduate Certificate/ Graduate Diploma/ Master or Doctoral degree		172	22.8
Financial position of owners		755		
	Finding it difficult or just getting along		263	34.8
	Reasonably comfortable		364	48.2
	Very comfortable or prosperous		128	17.0
State		752		
	Victoria		256	34.0
	New South Wales		180	23.9
	Queensland		147	19.5
	Other⁎		169	22.5
Area of residence		756		
	Rural/ Remote		101	13.4
	Suburbia/Semi-rural		614	81.2
	Inner city		41	5.4

⁎ Other includes Western Australia, South Australia, Tasmania, Australian Capital Territory, and Northern Territory.

### 3.1 Demographics of respondent's dogs and dog owner's practices, and perceptions regarding canine GI parasites

Table 2 details the demographics of respondent's dogs as well as their practices and perceptions regarding canine GI parasites. The majority of dog owners had one (51%) or two dogs (32%), mainly pure breeds (74%), belonging to the toy breed (22%) or working dogs kennel groups (21%). Most of dogs were allowed both indoors and outdoors (70%) and cohabited with other animals (67%), including other dogs and cats (94%), poultry and pigs (24%), and cattle and horses (8%).

**Table 2:** Australian dog owner's animals as well as practices and perceptions regarding canine gastrointestinal parasites (n = 759).

Variable name	Categories	N of respondents	Frequency	Percentage (%)
Dog's age		759		
	≤3 years		204	26.9
	4–6 years		185	24.4
	7–9 years		181	23.8
	>9 years		189	24.9
Dog's sex		758		
	Male neutered		291	38.4
	Female neutered		257	33.9
	Female intact		191	25.2
	Male intact		19	2.5
Dog's weight		755		
	≤9 kg		208	27.5
	10–17 kg		176	23.3
	18–28 kg		192	25.4
	>28 kg		179	23.7
Dogs per households		758		
	1		388	51.2
	2		245	32.3
	3		72	9.5
	>3		53	7.0
Dog's breed		750		
	Pure breed		553	73.7
	Mixed breed		197	26.3
Kennel groups		750		
	Toys		161	21.5
	Working dogs		155	20.7
	Terriers		106	14.1
	Gundogs		95	12.7
	Utility		41	5.5
	Hounds		40	5.3
	Non-Sporting		23	3.1
	Other^$^		129	17.2
Dog's housing		757		
	Both indoors and outdoors		528	69.7
	Indoors only		145	19.2
	Outdoors only (e.g., backyard or run)		84	11.1
Contact with other animals		758		
	Yes		506	66.8
	No		252	33.2
Species of contact⁎		506		
	Dogs and cats		476	94.1
	Poultry and pigs		120	23.7
	Cattle and horses		40	7.9
	Alpacas/sheep/goats		15	2.9
	Other^+^		30	5.9
Access to rodents, lizards, toads		758		
	No		364	48.0
	Yes		329	43.4
	Not sure		65	8.6
Frequency of walks		758		
	Daily		409	54.0
	At least twice a week		160	21.1
	At least once a week		48	6.3
	At least once a fortnight		46	6.1
	Not necessary, dog roams freely unsupervised (e.g., farm dog)		95	12.5
Removal of faeces during walks		727		
	Yes		628	86.4
	Sometimes		34	4.7
	No		28	3.9
	Not applicable		37	5.1
Disposal of faeces on the property		757		
	Daily		331	43.7
	At least twice a week		188	24.8
	At least once a week		125	16.5
	At least once a fortnight		66	8.7
	Never		47	6.2
Disposal method⁎		759		
	Place in garbage		613	80.8
	Bury into soil		82	10.8
	Flush down toilet		66	8.7
	*Re*-use as manure after composting		39	5.1
	Re-use as manure without composting		29	3.8
	Burn		11	1.4
	Other^£^		21	2.8
	Not applicable		26	3.4
Food⁎		759		
	Commercial food		649	85.5
	Home-made food incl. Table scraps		350	46.1
	Raw meat		294	38.7
	Raw vegetables		168	22.1
Frequency of vet visits		758		
	At least once per year		361	47.6
	At least once every 6 months		285	37.6
	Less than once per year		112	14.8
Perceived importance of dog parasites on human health		756		
	Not at all/ A little		208	27.5
	Moderately		198	26.2
	Very/Extremely		350	46.3
Perceived importance of parasites on dog health		756		
	Not at all/ A little		160	21.2
	Moderately		148	19.6
	Very/Extremely		448	59.3
Traveled/lived with dog interstate		756		
	No		617	81.6
	Yes		139	18.4
			
Traveled/lived with dog overseas		756		
	No		741	98.0
	Yes		15	2.0

+ Other includes exotics (reptiles), wildlife, turtles, fishes, rodents.

$ Other includes mixed breed or breed not recognized by the Australian National Kennel Council.

£ Other include throwing it outside, mowing, and green waste bin.

⁎ Multiple responses possible.

Most dog owners walked their dogs daily (54%) or at least twice a week (21%) and claimed to remove their dogs' faeces during walks (86%). Within their property, most of the dog owners disposed of their dogs' faeces daily (44%) or at least twice a week (25%) by placing it into the garbage (81%) or burying it into soil (11%). Dogs were fed commercial food (86%), home-made food or table scraps (46%), raw meat (39%) and raw vegetables (22%).

Most of owners visited a veterinarian at least once per year (48%) or once every six months (38%). Just over a half of dog owners considered parasites as very or extremely important for their dog's health (59%) and less than a half as very or extremely important for human health (46%).

Overall, 90% (678/757) of dog owners stated that they deworm their dogs (Table 3). The majority of dog owners treated their dogs for canine GI parasites every month (37%) or every 2–3 months (34%), and all year round (87%). Only the 65% (440/678) of dog owners who claim to deworm their dogs were also able to state the name of the product of choice. The active compounds most commonly used by dog owners to deworm their dogs were afoxolaner/milbemycin oxime (20%), followed by imidacloprid/moxidectin (13%), and praziquantel/pyrantel pamoate/febantel (12%) (Table 3). In total, 24% (182/745) of dog owners did not administer any effective parasite prophylaxis, 48% (356/745) did not to follow best prophylaxis guidelines, and only 28% (207/745) followed best parasite prophylaxis practice.

**Table 3:** Australian dog owner's deworming practices and active compounds most commonly used to treat for canine gastrointestinal parasites.

Variable name	Categories	N of respondents	Frequency	Percentage (%)
Deworm their dogs		757		
	Yes		678	89.6
	No		67	8.9
	Don't know		12	1.6
Frequency of treatment		678		
	Every month		252	37.2
	Every 2–3 months		229	33.8
	Every 4–6 months		133	19.6
	Every 7–12 months		35	5.2
	Less than every 12 months		14	2.1
	I don't administer treatment for intestinal parasites		15	2.2
Duration of treatment		678		
	All year round		587	86.6
	During the warmer months only		36	5.3
	Only when my dog is sick		17	2.5
	Only when parasites are seen in my dog's stool or vomit		16	2.4
	Other⁎		22	3.2
Active compound		440		
	Afoxolaner/milbemycin oxime		86	19.5
	Imidacloprid/moxidectin		55	12.5
	Praziquantel/pyrantel pamoate/febantel		52	11.8
	Afoxolaner		47	10.7
	Milbemycin oxime/praziquantel		41	9.3
	Pyrantel embonate/oxantel embonate/praziquantel		33	7.5
	Fluralaner		22	5.0
	Milbemycin oxime/lufenuron		19	4.3
	Oxibendazole/praziquantel		19	4.3
	Fipronil		18	4.1
	Selamectin		12	2.7
	Sarolaner		8	1.8
	Milbemycin oxime/lufenuron/praziquantel		6	1.4
	Spinosad/milbemycin oxime		6	1.4
	Ivermectin/pyrantel pamoate		4	0.9
	Levamisole/niclosamide		3	0.7
	Sarolaner/ moxidectin/pyrantel		3	0.7
	Imidacloprid/ flumethrin		2	0.5
	Abemectin/oxibendazole/praziquantel		1	0.2
	Praziquantel/febantel		1	0.2
	Pyrantel		1	0.2
	Pyrantel embonate/fenbendazole/praziquantel		1	0.2
Suitability of parasiticides protocol		745		
	Inappropriate prophylactic treatment		356	47.8
	Appropriate prophylactic treatment		207	27.8
	No prophylactic treatment		182	24.4

⁎ Other includes when they remembered or done by the veterinarians during visits.

### 3.2 Association between suitability of parasiticide protocol used and respondent's demographics

Univariable analysis revealed that gender, education, financial position of owners, area of residence, dog's age, frequency of vet visits, and perceived importance of parasites on dog health, were all significantly associated (*p*-value <0.25) with suitability of parasiticide protocol used (Table S2). None of these variables were highly correlated and therefore all were included in the multivariable logistic regression analysis.

The final multivariable model is shown in Table 4. Financial position of owners, and frequency of vet visits remained significant with suitability of parasiticide protocols used. The odds of administrating a correct anthelmintic prophylaxis were higher in those who had a better financial situation and attended the veterinarian at least once a year or once every six months. All the variables significant at the univariable analysis were confirmed to have a non-confounding effect. The final multivariable model was statistically significant (*p* < 0.001). Model fit to the data was assessed as being adequate (Pearson goodness-of-fit test *p* = 0.61, deviance goodness-of-fit test p = 0.61) and the assumption of proportional odds was fulfilled (Score test *p* = 0.36).

**Table 4:** Multivariable ordinal regression analysis of variables associated with suitability of parasiticide protocols used based on survey responses of Australian dog owners (*n* = 742).

Variable	Category	Estimate	Std. Error	Odds ratio (95% Confidence interval)	p-value
Intercept low		−0.83	0.25	0.43 (0.27–0.71)	
Intercept medium		1.32	0.25	3.75 (2.28–6.17)	
Financial position of owners					0.004
	Finding it difficult or just getting along	Ref.			
	Reasonably comfortable	0.43	0.15	1.54 (1.14–2.09)	
	Very comfortable or prosperous	0.58	0.21	1.79 (1.2–2.68)	
Frequency of vet visits					0.002
	At least once every 6 months	0.7	0.22	2.02 (1.32–3.09)	
	At least once per year	0.72	0.21	2.05 (1.36–3.1)	
	Less than once per year	Ref.			

## 4 Discussion

This study investigated Australian dog owners' demographics and perceptions towards canine GI parasites and factors that influence the likelihood of administering an effective anthelmintic prophylactic treatment. Less than one third of dog owners (28%) followed best prophylaxis guidelines consisting of administering an effective anthelmintic on a monthly basis, all year round. Most dog owners administered a prophylactic treatment at an inappropriate frequency (48%) or did not treat for canine GI parasites at all (24%). Similar to observations in Europe [13,18,22], we found a high proportion of pet owners administering anthelmintic prophylaxis at irregular intervals, often resulting in inefficient treatment protocols.

TROCCAP guidelines recommends monthly deworming of adult dogs all year round [20]. Similarly, CAPC guidelines also recommends the year-round broad-spectrum treatment of adult dogs with products effective against canine GI parasites. However, it also states that if a year-round protection cannot be maintained, quarterly treatment of adult dogs would suffice [19]. We believe that this information might be misinterpreted by veterinarians who might believe they are complying with guidelines by recommending quarterly deworming of adult dogs. In addition, many web-based resources freely accessible online to veterinarians and dog owners recommend three-monthly deworming of adult dogs (https://vitapet.com/au/vitapet-central/articles/puppy-worming-schedule/, https://www.vetwest.com.au/, https://miramarvet.com.au/worming-what-are-your-options/). As a likely consequence, we found a high proportion of dog owners in this study administering a prophylactic treatment every 2–3 months (34%).

However, owing to the high risk of exposure of dogs and humans to canine GI parasites in Australia including hookworms, and *Strongyloides* spp., the first and third most common canine soil-transmitted helminths recently recovered from parks in Australia [9], and their short prepatent period of one to three weeks [[23], [24], [25]], it is important that dogs in Australia are maintained on a monthly protection against canine GI parasites in order to reduce environmental contamination and risk of zoonotic transmission. Veterinarians play an important role in the control of canine parasitic diseases through educating and encouraging dog owners into following regular monthly protocol treatments effective against canine GI parasites as recommended in various guidelines.

Frequency of veterinarian visits was the primary factor that motivated dog owners to adhere to best practices for deworming their pets. Attending veterinary consults at least annually or once every six months was significantly associated with following best practice deworming guidelines. As veterinarians are the main source of knowledge for pet owners [1,17,26], it is likely that owners paying frequent visits to the veterinarian were better informed on the importance of parasites and best-practice guidelines for their treatment and control. Furthermore, regular veterinary visits, might also favour the establishment of a long-term client-veterinarian relationship which in turn, is associated with a higher level of owner compliance with preventive veterinary care [27,28].

Veterinarians have a duty towards the community to educate owners to follow best prophylaxis guidelines, including advising on the use of effective compounds for treatment, and raising their awareness on the risks canine parasitic diseases pose to their animals and themselves [29]. However, in Australia, just under half of veterinarians recently surveyed, correctly identified appropriate canine deworming protocols [30]. Moreover, Australian veterinarians scored poorly on overall knowledge on the important canine GI parasites occurring within their practice area [30]. Therefore, continuing veterinary education on companion animal parasitology plays a key role not only for improving standards of veterinary knowledge, but also ensuring that owners are also educated appropriately on the importance of following best practice guidelines for the control of GI parasites in their pets.

Only 65% of respondents who claim to administer a prophylactic treatment against canine endoparasites were also able to state the name of the product used showing an overall low familiarity of dog owners in Australia towards parasiticide treatments. It is plausible that the unfamiliarity with the product used might result in inappropriate or ineffective treatment due to administration errors such as underdosing, inappropriate dosing intervals or use of ineffective compounds. Client education on the most appropriate product and method of administration, as well as the use of simplified treatment regimens (e.g., palatable chews, use of endectoparasiticides to minimise the number of products needed) may help to ensure efficacy of treatments.

>20% of respondent dog owners who claimed to administer a prophylactic treatment against canine endoparasites and were able to state the name of the parasiticide, used products that were only effective against ectoparasites (i.e., afoxolaner, fluralaner, sarolaner, fipronil, and imidacloprid). While it is possible that this is a result of veterinarians failing to prescribe an appropriate endoparasiticide, we consider this unlikely. Rather, we consider this finding most likely due to the ability of owners to purchase parasiticides “over the counter” either over the internet, or in person in supermarkets and pet shops. In this scenario, veterinarians unfortunately have little input on advising dog owners on appropriate deworming products and regimens. Such a finding does however reinforce the importance of veterinarians proactively discussing parasite control at every available opportunity with their clients.

The financial position of owners was also significantly associated with dog owners following best practice GI parasite control guidelines. Respondents in a very comfortable or prosperous economic position were almost two times more likely to effectively deworm their dogs compared to those who were just ‘getting along’ or ‘finding it difficult’. Owners with a higher disposable income are more likely to be able to afford purchasing frequent anthelmintic treatments for their dogs. Affordability was also recently demonstrated as the main barrier for veterinarians to deliver best practice in terms of diagnosis, treatment, and control of canine parasitic diseases [13,30,31]. A recent national survey demonstrated that parks in Australia located within postcodes with lower socioeconomic indices were more likely contaminated with canine helminths [9]. It therefore emerges that socioeconomic factors play a key role in influencing the prevalence of canine GI parasites and the risk they pose to other dogs and humans. Similarly, in a recent large-scale survey on companion animal parasites in Asia, an increased life expectancy in humans and neutering status of animals, which are indicative of higher living standards through access to education and health care for both humans and animals, had a significant effect on the exposure to zoonotic parasites in dogs and cats [5]. Thus, veterinarians should ensure that efforts are made to specifically target clients in lower socioeconomic areas with respect to educating them on responsible dog ownership and on effective non-cost dependent management practices (e.g., prompt removal of faeces from environment, avoid feeding raw meat) that will reduce risks of parasitic infections in their pets and themselves, even when affordability may limit their ability to administer anthelmintics at the frequency recommended-.

A significant proportion of respondents fed their dogs raw meat or vegetables, a trend that has been growing on a global level in recent years [22,32,33] owing to owner belief that a raw meat diet is safe and beneficial for their dogs with claimed improvements on the coat, muscle mass, and oral health [32]. However, feeding raw food, might also expose dogs to a plethora of parasites including *Neospora caninum*, *Cryptosporidium* spp., *Sarcocystis* spp., *Cystoisospora* spp., *Hammondia hammondi*, *T. canis, A. caninum*, *S. erinacei*, *Taenia* spp. and *Echinococcus* spp. Infections with some of these parasites may not only impact the health of dogs, but are also zoonotic [[34], [35], [36]]. As such, there is again a need for veterinarians to discuss dietary needs of dogs and make recommendations towards commercial food and/or cooked meat to reduce the risk of exposure to GI parasites in dogs and humans.

The majority of dog owners did not properly dispose of their dog faeces within their properties, with less than half of respondents (44%) removing their dog's faeces on a daily basis. Furthermore, a small, yet significant proportion of owners did not always remove their dog's faeces during walks and admitted to disposing faeces by burying it into soil, or by re-using it as manure without composting. Some species of canine GI parasites passed into faeces are immediately infective (e.g., *Giardia* and *Echinococcus* spp.), thus posing an immediate risk of transmission to owners and their families either directly, through close contact with the animal or indirectly through the environment. Soil transmitted helminths (e.g., *Strongyloides*, hookworms, *T. canis*), require between a few days to a few weeks to become infective and may survive in the environment from months to years [[37], [38], [39]]. Prompt and safe disposal of dog faeces on a daily basis is by far the most important management practice owners can adopt to minimise the risk of parasite transmission via their dogs [40]. To minimise the environmental contamination with species of canine GI parasites, measures to facilitate the removal of faeces such as the provision of disposal bins and bags should be encouraged in communal locations and leash-control and faecal clean-up laws should also be enforced by local authorities [9].

This study has some limitations and is prone to selection bias as only responses from 759 dog owners were included in the analyses compared to the estimated 4.8 million of households with at least one dog in Australia [1]. Thus, owners with a greater interest in the topic might have been more likely to participate and complete the survey compared to those who were less interested. It could also be assumed that owners interested in this topic are more likely to consider canine parasites as a threat for their dogs' health and hence, more inclined to follow effective control protocols. Furthermore, most of the respondents were females, in a comfortable or prosperous financial position, and had regular access to veterinary care. Previous research indicated that female respondents, who are often primary caregivers in households, might be more likely to deworm their dogs frequently [41]. Thus, participants in this study could be regarded as ‘best cases’ rather than representative of Australian dog owners in general. Nonetheless, complacency and non-compliance with best management and prophylaxis guidelines around canine GI parasites were common even in this group, highlighting the importance of education and awareness campaigns Interestingly, the distribution of respondent dog owners in this study largely reflects the distribution of pet dogs in Australia; for instance, 79% of questionnaires stemmed from Victoria, New South Wales, Australian Capital Territory, and Queensland where nearly 80% of the total population of pet dogs in Australia is estimated to reside [21]. Further, this study includes dog owners of different ages, levels of education and financial positions. However, this study is based on self-reported and not observed behaviour, meaning that response bias could be present. Knowing the researchers were veterinarians, it is possible that participants might have overstated their practices and behaviour in regard to what they assumed to be desirable and might be viewed as responsible dog ownership. Consequently, the percentage of owners following best practice in Australia regarding canine GI parasites might even be lower than reported in this study. However, to minimise this risk and ensure reliability of responses, questionnaires were completed anonymously.

## 5 Conclusions

Overall, this study demonstrates that a vast majority of dog owners in Australia are not following best prophylaxis guidelines for the treatment and control of canine endoparasites. Although most owners administer anthelmintic drugs to their dogs, this often occurs at irregular intervals. As a consequence the treatment may be inefficient in the medium to long term. Furthermore, there was a large proportion of dog owners that did not follow best management practices, admitting to not properly disposing of their dog's faeces and feeding their dogs raw food. These practices are exposing themselves and their dogs to an increased risk of infection. Veterinarians as a trusted information source should therefore address these topics, raise awareness of the threats inherent to canine parasitic diseases, and finally encourage owners to follow best prophylaxis and management guidelines. Local authorities are also called upon to promote and enforce dog sanitation laws to minimise environmental contamination with canine GI parasites. Altogether this would lay the foundation for an improved One Health intervention targeting canine gastrointestinal parasites in Australia.

## Funding

This research study was funded by Boehringer Ingelheim Animal Health Australia.

## CRediT authorship contribution statement

**Luca Massetti:** Methodology, Investigation, Formal analysis, Writing – original draft. **Rebecca J. Traub:** Conceptualization, Supervision, Writing – original draft, Funding acquisition. **Louise Rae:** Resources, Writing – review & editing. **Vito Colella:** Supervision, Writing – review & editing. **Lara Marwedel:** Resources, Writing – review & editing. **Phillip McDonagh:** Resources, Writing – review & editing, Funding acquisition. **Anke Wiethoelter:** Conceptualization, Methodology, Validation, Supervision, Writing – original draft.

## Declaration of Competing Interest

This research study was funded by Boehringer Ingelheim Animal Health Australia. Louise Rae, Lara Marwedel, and Phillip McDonagh are employees at Boehringer Ingelheim Animal Health Australia. Luca Massetti is supported by a 10.13039/501100001782University of Melbourne, Australia, Research Scholarship.

## Data Availability

Data will be made available on request.

## Appendix A Supplementary data

S1. Questionnaire for dog owners. Image 1
