# Supplementary material for: Canine gastrointestinal parasites perceptions, practices, and behaviours: A survey of dog owners in Australia
Source: One Health. 2023 Jun 16;17:100587. doi: 10.1016/j.onehlt.2023.100587 (PMC10320617; doi:10.1016/j.onehlt.2023.100587)
Supplement: Supplementary file 1 — S1. Questionnaire for dog owners. [file mmc1.docx]

# Supplementary material

S1. Questionnaire for dog owners.

| 1. Are you a dog owner?  Yes  No | | |
| --- | --- | --- |
| 2. What’s the age of your dog? (If under a year please state in months)  ………………………………………………….. (in years) | | |
| 3. What’s your dog’s breed?  ………………………………………………… | | |
| 4. What sex is your dog?  Male intact  Female intact  Male neutered  Female neutered | | |
| 5. What’s your dog’s weight?  ………………………………………………… (kilograms) | | |
| 6. In the last 12 months, has your dog shown any of the following signs? | | |
| Exercise intolerance | | Yes  No |
| Weight loss | | Yes  No |
| Cough | | Yes  No |
| Difficult breathing | | Yes  No |
| Vomiting | | Yes  No |
| Diarrhoea | | Yes  No |
| Skin diseases | | Yes  No |
| 7. Does your dog have contact with other animals within your home / property?  Yes  No   \| Animal species \| How many? \| Animal species \| How many? \| \| --- \| --- \| --- \| --- \| \| Dogs \|  \| Pigs \|  \| \| Cats \|  \| Cattle \|  \| \| Horses \|  \| Birds incl. Poultry \|  \| \| Alpacas/Sheep/  Goats \|  \| Other, please specify: …………………………………… \|  \| | | |
|  | | |
| 8. On average, how often do you take your dog to the veterinarian?  At least once every 6 months 🞎 At least once per year  At least once every two years  Less than once every two years 🞎 Only when sick | | |
| 9. Where does your dog spend most of its time?  Indoors only  Outdoors only (e.g. backyard or run)  Both indoors and outdoors | | |
| 10. How often do you take your dog out for a walk?  Daily  At least twice a week  At least once a week  At least once a fortnight  Not necessary, dog roams freely unsupervised (e.g., farm dog) GO TO Q12 | | |
| 11. Do you remove and dispose of your dog’s faeces during walks?  Yes  No  Sometimes  Not applicable | | |
| 12. How often do you dispose of your dog’s faeces within the confines of your property?  Daily  At least twice a week  At least once a week  At least once a fortnight  Never | | |
| 13. What method do you use to dispose of your dog’s faeces? Please, tick all that apply.  Place in garbage  Bury into soil  Burn  Flush down toilet  Re-use as manure without composting  Re-use as manure after composting  Not applicable (I don’t dispose of my dog’s faeces)  Other, please specify: ……………………………………...…………………………………… | | |
| 14. Do you treat your dog for intestinal parasites or worms?  Yes  No  Don’t know.  IF ‘Yes’, please specify product name(s) ……………..……………………………………………  IF ‘No’ or ‘Don’t’ know’, GO TO Q17. | | |
| 15. When do you treat your dog for intestinal parasites?  All year round  During the warmer months only  Only when my dog is sick  Only when parasites are seen in my dog’s stool or vomit  Other, please specify: …………………………………………...…………………………… | | |
| 16. On average, how often do you administer treatment for intestinal parasites?  Every month  Every 2-3 months  Every 4-6 months  Every 7-12 months  less than every 12 months  I don’t administer treatment for intestinal parasites | | |
| 17. Do you treat your dog for external parasites (e.g., fleas, ticks)?  Yes  No  Don’t know  IF ‘Yes’, please specify product name(s): …………………………………………………………  If ‘No’ or ‘Don’t know’ GO TO Q20. | | |
| 18. If yes, when do you treat your dog for external parasites (e.g., fleas and ticks)?  All year round  During the warmer months only  Only when my dog is sick  Only when parasites are seen on my dog  Other, please specify ……………...……………………………………………………………… | | |
| 19. On average, how often do you administer treatment for external parasites?  Every week  Every fortnight  Every month  Every 2-4months  Every 5- 6-months  Every 7-12-months  Less than every 12 months  I don’t administer treatment for external parasites. | | |
| 20. What do you feed your dog? Please tick all that apply.  Commercial dog food 🞎 Homemade cooked food (incl. table scraps)  Raw vegetables  Raw meat (including bones and chicken necks)  Other, please specify: …………………………………………………………………………….. | | |
| 21. Does your dog have access to areas that may harbor rodents, lizards, or toads?  Yes  No  Not sure | | |
| 22. Have you ever observed the following on your dog? | | |
| Ticks | Yes  No | |
| Fleas | Yes  No | |
| ‘Worms’ or worm segments in their stool | Yes  No | |
| ‘Worms’ in their vomit | Yes  No | |
| Worms’ or worm segments on their coat | Yes  No | |
| Scooting or rubbing their bottoms | Yes  No | |
| 23. Has your dog ever travelled or lived in another state of Australia?  Yes  No  If ‘Yes’ select all the states and territories in which your dog has travelled / lived.  New South Wales  Victoria  Queensland  South Australia  Western Australia  Tasmania  Northern Territory  Australian Capital Territory | | |
| 24. Has your dog ever lived or travelled overseas?  Yes  No  If ‘Yes’, please specify country(ies): ……………………………………………………………… | | |
| 25. How significant do you think parasites are to your dog's health?  Not at all  A little  Moderately  Very  Extremely | | |
| 26. How significant do you think parasites of your dog are for your own health?  Not at all  A little  Moderately  Very  Extremely | | |
| 27. Do you know whether external parasites may transmit blood-borne diseases to your dog   \| Ticks \| No  Yes, but I am unfamiliar with the disease(s) they transmit  Yes, and I can describe or name at least one disease they transmit. Please specify: …………………………………………………………………………………… \| \| --- \| --- \| \| Fleas \| No  Yes, but I am unfamiliar with the disease(s) they transmit  Yes, and I can describe or name at least one disease they transmit. Please specify: …………………………………………………………………………………… \| \| Mosquitoes \| No  Yes, but I am unfamiliar with the disease(s) they transmit  Yes, and I can describe or name at least one disease they transmit. Please specify: …………………………………………………………………………………… \| | | |
| 28. How likely do you think it is that fleas transmit diseases between animals and humans?  Not at all  A little  Moderately  Very  Extremely | | |
| 29. How likely do you think it is that ticks transmit diseases between animals and humans?  Not at all  A little  Moderately  Very  Extremely | | |
| 30. How likely do you think it is that mosquitoes transmit diseases between animals and humans?  Not at all  A little  Moderately  Very  Extremely | | |
| 32. What’s your age group (in years)?  18-24 🞎 25-34  35- 44  45-54  55- 64  65 and over | | |
| 33. What’s your gender?  Male 🞎 Female  Other | | |
| 34. What is the highest level of education you have completed?  Year 11 or below (including Certificate I/II)  Year 12  Certificate III/IV  Diploma or Advanced Diploma  Bachelor Degree  Graduate Certificate or Graduate Diploma  Master Degree  Doctoral Degree | | |
| 35. What is your postcode of residence?  ………………………………………………………………………………………………………… | | |
| 36. How would you best describe the area in which you reside?  Inner city 🞎 Suburbia  Semi-rural  Rural | | |
| 37. Given your current needs and financial responsibilities, would you say that you and your household are:  Finding it difficult  Just getting along  Reasonably comfortable  Very comfortable  Prosperous | | |

## Table S2. Univariable ordinal regression analysis of variables associated with suitability of parasiticide protocols used based on survey responses of Australian dog owners (n= 745).

| **Variable** | **Category** | **Estimate** | **Std. Error** | **Odds ratio (95% Confidence interval)** | **p-value** |
| --- | --- | --- | --- | --- | --- |
| Gender |  |  |  |  | 0.032 |
|  | Male | Ref. |  |  |  |
|  | Female | 0.33 | 0.15 | 1.39 (1.03-1.88) |  |
| Age |  |  |  |  | 0.936 |
|  | 18-34 years | 0.02 | 0.18 | 1.02 (0.72-1.45) |  |
|  | 35- 54 years | 0.06 | 0.17 | 1.06 (0.76-1.47) |  |
|  | ≥55 years | Ref. |  |  |  |
| Education |  |  |  |  | 0.018 |
|  | Certificate IV or below | Ref. |  | . |  |
|  | Diploma/ Advanced Diploma/ Bachelor’s degree | 0.11 | 0.16 | 1.11 (0.82-1.52) |  |
|  | Graduate Certificate/Graduate Diploma/Master or Doctoral degree | 0.5 | 0.18 | 1.66 (1.16-2.36) |  |
| Financial position of owners |  |  |  |  | 0.001 |
|  | Finding it difficult or just getting along | Ref. |  |  |  |
|  | Reasonably comfortable | 0.45 | 0.15 | 1.57 (1.16-2.12) |  |
|  | Very comfortable or prosperous | 0.65 | 0.20 | 1.91 (1.28-2.85) |  |
| State |  |  |  |  | 0.293 |
|  | New South Wales | Ref. |  |  |  |
|  | Victoria | 0.35 | 0.19 | 1.41 (0.98-2.04) |  |
|  | Queensland | 0.13 | 0.21 | 1.13 (0.75-1.71) |  |
|  | Other | 0.13 | 0.2 | 1.14 (0.77-1.69) |  |
| Area of residence |  |  |  |  | 0.058 |
|  | Rural/ Remote | Ref. |  |  |  |
|  | Suburbia/Semi-rural | -0.01 | 0.2 | 0.99 (0.67-1.46) |  |
|  | Inner city | 0.71 | 0.35 | 2.03 (1.03- 4) |  |
| Dog’s age |  |  |  |  | 0.174 |
|  | ≤3 years | 0.35 | 0.19 | 1.42 (0.98-2.06) |  |
|  | 4-6 years | 0.34 | 0.19 | 1.4 (0.96-2.06) |  |
|  | 7-9 years | 0.09 | 0.20 | 1.1 (0.75-1.61) |  |
|  | >9 years | Ref. |  |  |  |
| Dog’s sex |  |  |  |  | 0.171 |
|  | Male intact | Ref. |  |  |  |
|  | Female intact | 0.17 | 0.45 | 1.18 (0.49-2.86) |  |
|  | Male neutered | 0.37 | 0.44 | 1.44 (0.6-3.45) |  |
|  | Female neutered | 0.54 | 0.45 | 1.72 (0.72-4.12) |  |
| Dog’s weight |  |  |  |  | 0.901 |
|  | ≤9 kg | Ref. |  |  |  |
|  | 10-17 kg | 0.06 | 0.19 | 1.07 (0.73-1.55) |  |
|  | 18-28 kg | 0.10 | 0.19 | 1.1 (0.76-1.6) |  |
|  | >28 kg | 0.14 | 0.19 | 1.15 (0.79-1.68) |  |
| Dogs per household |  |  |  |  | 0.437 |
|  | 1 | Ref. |  |  |  |
|  | 2 | 0.11 | 0.15 | 1.11 (0.82-1.5) |  |
|  | 3 | -0.15 | 0.24 | 0.86 (0.54-1.38) |  |
|  | >3 | 0.36 | 0.28 | 1.43 (0.83-2.48) |  |
| Dog’s breed |  |  |  |  | 0.489 |
|  | Pure breed | Ref. |  |  |  |
|  | Mixed breed | 0.11 | 0.16 | 1.12 (0.82-1.52) |  |
| Frequency of vet visits |  |  |  |  | <0.001 |
|  | At least once every 6 months | 0.76 | 0.21 | 2.13 (1.4-3.24) |  |
|  | At least once per year | 0.76 | 0.21 | 2.14 (1.42-3.23) |  |
|  | Less than once per year | Ref. |  |  |  |
| Perceived importance of dog parasites on human health |  |  |  |  | 0.570 |
|  | Not at all/ A little | Ref. |  |  |  |
|  | Moderately | 0.15 | 0.19 | 1.17 (0.81-1.68) |  |
|  | Very/Extremely | 0.17 | 0.17 | 1.19 (0.85-1.65) |  |
| Perceived importance of parasites on dog health |  |  |  |  | 0.052 |
|  | Not at all/ A little | Ref. |  |  |  |
|  | Moderately | 0.26 | 0.22 | 1.3 (0.85-1.98) |  |
|  | Very/Extremely | 0.43 | 0.18 | 1.53 (1.08-2.17) |  |
